# Rectal Colonization by Drug Resistant Bacteria in Nursing Home Residents in Crete, Greece

**DOI:** 10.3390/tropicalmed6030123

**Published:** 2021-07-05

**Authors:** Aikaterini Moschou, Petros Ioannou, Eleni Moraitaki, Dimitra Stafylaki, Sofia Maraki, George Samonis, Diamantis P. Kofteridis

**Affiliations:** 1 Department of Internal Medicine, University Hospital of Heraklion, Heraklion, PC 71110 Crete, Greece; kmoschou@hotmail.com (A.M.); samonis@med.uoc.gr (G.S.); kofteridisd@hotmail.com (D.P.K.); 2 Department of Clinical Microbiology, University Hospital of Heraklion, PC 71110 Crete, Greece; ra8820027@yahoo.com (E.M.); stafylaki.dimitra@gmail.com (D.S.); sofiamaraki@yahoo.gr (S.M.)

**Keywords:** multi-drug resistance, ESBL, MDR, nursing homes, long-term care

## Abstract

(1) Background: In an area with a high prevalence of multi-drug resistant Gram-negative bacteria (MDR-GNB), we investigated the colonization of nursing home residents by such organisms. (2) Methods: A point prevalence study was performed in six nursing homes of the Heraklion area on the island of Crete. A rectal swab was taken and cultured from each participant, while additional risk factors such as recent hospitalization or antimicrobial usage were recorded and evaluated. (3) Results: A total of 137 nursing home residents were included in the study. Their mean age was 82.1 years and 19.7% were males. In total, cultures yielded 255 GNB; *E. coli*, *K. pneumoniae* and *P. aeruginosa* were the most common. Among the microorganisms cultured, 17.6% had the extended-spectrum beta-lactamase phenotype, while 18% were MDR. A statistically significant association was found between recent antimicrobial use and colonization by MDR-GNB; (4) Conclusions: Colonization by MDR-GNB was found to be highly prevalent in nursing home residents. Recent antimicrobial use was associated with MDR-GNB carriage.

## 1. Introduction

Improvement of medical services and lifestyle in the Western world has led to a significant increase in life expectancy, resulting in ageing of the population. Older people often need long term care that is hard to offer at home, thus long-term care facilities (LTCFs) have become important.

Multi-drug resistant (MDR) Gram-negative bacteria (GNB) are becoming increasingly prevalent [1,2]. Treatment of MDR-GNB infections can be problematic, since inappropriate empirical initial antimicrobial treatment often leads to increased hospital stay, costs, morbidities and mortality [3]. Colonization by MDR-GNB carries a high risk of infection [4]. Patients with recent healthcare or hospital contacts and patients in LTCFs are more commonly colonized by such organisms [5,6]. A recent systematic review has shown that 20% of LTCF residents were colonized by ESBL-producing microorganisms, and that these individuals were more likely to have had a recent healthcare facility contact or history of antimicrobial use [6]. Colonization of LTCF residents has also been shown in other studies to involve other pathogens, such as methicillin resistant *Staphylococcus aureus*, or carbapenemase-resistant *Enterobacteriaceae* and vancomycin-resistant *Enterococcus* [7,8,9,10,11], with evidence suggesting clonal dissemination in many cases [7].

Identification of increased prevalence of MDR-GNB in LTCFs is crucial, since these microorganisms could represent a niche for further dissemination to the community, and even further increase antimicrobial resistance [12]. Improvement can be achieved through targeted interventions by implementation of infection control measures and antimicrobial stewardship practices in order to reduce horizontal spread of the resistant microorganisms and antimicrobial usage, respectively [12,13,14].

The aim of this study was to investigate the prevalence of MDR-GNB colonization among nursing home residents in an area with a high hospital prevalence of MDR-GNB, such as the island of Crete, Greece.

## 2. Materials and Methods

### 2.1. Study Duration, Setting, Study Participants and Population

This is a point prevalence study performed from May to August 2017 in six nursing homes of the Heraklion area on the island of Crete. All six participating nursing homes (NH1, NH2, NH3, NH4, NH5 and NH6) were private and their capacity was 78, 75, 70, 118, 60 and 55 beds, respectively. Rectal swabs of all eligible participants, with an age ≥ 65 years, were obtained and consequently cultured (only once from each participant). Residents with infections were excluded. The study duration for each LTCF was two weeks.

Each of the 137 participants was assessed for additional risk factors using a questionnaire relating to urinary catheters, comorbidities, hospital admission during the preceding three months and antimicrobial usage for the same period. Information was gathered with interviews with the residents and by studying the notes of their medical records. Isolates were characterized as MDR if they were non-susceptible to at least one agent in at least three antimicrobial categories, as previously described [15].

The study protocol, complying with the Helsinki Declaration, was approved by the Ethics Committee of the University Hospital of Heraklion, Crete, Greece, and informed consent was obtained from each participant.

### 2.2. Microbiological Analysis

Rectal swabs were investigated for the presence of GNB with direct culturing on MacConkey agar. After 24 to 48 h of incubation at 36 °C under aerobic conditions, Gram-negative colonies were subcultured onto MacConkey agar to isolate pure colonies. Genus and species identification was performed using standard biochemical methods, and the VITEK2 automated system (BioMérieux, Marcy l’ Etoile, France). Antimicrobial susceptibility testing was performed using the disk diffusion method and the VITEK2 automated system. The Clinical and Laboratory Standards Institute (CLSI) breakpoints were used for the interpretation of results of all antimicrobial agents except for tigecycline and colistin [16]. The MICs (minimum inhibitory concentrations) of tigecycline and colistin were interpreted following the U.S. Food and Drug Administration (FDA) and the European Committee on Antimicrobial Susceptibility Testing (EUCAST) criteria [17,18].

The double-disk synergy test was used for preliminary classification of the isolates as ESBL (extended-spectrum β-lactamase) producers. The synergistic activity of CA (clavulanic acid) with both ceftazidime and cefotaxime was confirmed by means of E-test special strips (BioMérieux) containing ceftazidime/ceftazidime plus CA and cefotaxime/cefotaxime plus CA [16].

### 2.3. Statistical Analysis

Categorical variables are presented as numbers (%), while continuous variables are presented as medians (interquartile range, IQR). Categorical variables were assessed by Pearson’s Chi-square test or Fisher’s exact test and continuous variables were assessed by Student’s *t*-test for normally distributed variables and the Mann–Whitney U-test for non-normally distributed variables. All tests were two-tailed and *p*-values < 0.05 were considered to be significant. A univariate logistic regression analysis was conducted to identify factors associated with colonization by ESBL-producing microorganisms or MDR-GNB. The above-mentioned statistics were calculated with GraphPad Prism 6.0 (GraphPad Software, Inc., San Diego, CA, USA). A multivariate logistic regression analysis was conducted to evaluate the effect of factors that were previously identified in the univariate analysis model, as factors with a *p*-value lower than 0.1. Multivariate analysis was performed using SPSS version 23.0 (IBM Corp., Armonk, NY, USA).

## 3. Results

A total of 137 LTCF residents, with an age ≥ 65 years, were included in the study. More specifically, 12 out of 78 residents from NH1, 34 out of 75 from NH2, 28 out of 70 from NH3, 33 out of 118 from NH4, 25 out of 60 from NH5 and 5 out of 55 from NH6 agreed to participate in the study. Their mean age was 82.1 years and 19.7% were males (27 residents). The most common underlying medical conditions were neurological diseases (dementia and Parkinson’s disease) in 78.8% (108 residents), arterial hypertension in 43.8% (60), diabetes mellitus in 19.7% (27) and congestive heart failure in 7.3% (10). Urinary catheter was present in 5.1% (7 residents), and 3.6% (5) had decubitus ulcers. A Charlson score of ≥3 was noted in 11.7% (16). Within the last 3 months, 8.8% (17) had received antimicrobials while 5.1% (7) had been hospitalized during the same period. In total, cultures yielded 255 isolates; *E. coli*, *K. pneumoniae* and *P. aeruginosa* were the most common, representing 64.3%, 11.8% and 5.5% of the isolates, respectively (164, 30 and 14 isolates). Table 1 shows the gender and species of the isolated microorganisms.

Among the isolated organisms 45 (17.6%) had the extended-spectrum beta-lactamase (ESBL) phenotype, while 46 (18%) were MDR. These results had shown that 36 residents (26.3%) were colonized by ESBL producing microorganisms and 33 (24.1%) by MDR-GNB, respectively. All individuals with MDR-GNB were colonized by ESBL-producing microorganisms. Colonization by MDR-GNB differed among different nursing homes, and more specifically, MDR-GNB positive samples were detected in 41.7% (5 out of 12) residents of NH1, 8.8% (3 out of 34) residents of NH2, 21.4% (6 out of 28) residents of NH3, 6.1% (2 out of 33) residents of NH4, 48% (12 out of 25) residents of NH5 and 100% (5 out of 5) residents of NH6. Subgroup comparison between male and female individuals (Table S1), those with and without hospitalization within the last three months (Table 2), those with and without antimicrobial use within the last three months (Table 3), those with and without colonization by MDR-GNB (Table 4) and those with and without colonization by ESBL-producing microorganisms (Table 5) was performed in order to identify statistically significant differences in terms of gender, age, duration of stay in the LTCF, Charlson Comorbidity Index, presence of diabetes, a urinary catheter or a neurological disorder and antimicrobial use or hospitalization within the last three months and colonization by MDR-GNB or ESBL-producing microorganisms. Contingency analysis identified a statistically significant association between antimicrobial use and hospitalization within the last three months; recent antimicrobial use was associated with a higher possibility of colonization by MDR-GNB and more specifically with microorganisms with the ESBL phenotype. Furthermore, the presence of a urinary catheter was significantly more frequent in individuals colonized by MDR-GNB and ESBL-producing microorganisms. Table S1 shows the characteristics of LTCF residents in regards to gender. In specific, male LTCF residents had a statistically significant higher duration of LTCF stay, but did not differ in terms of other characteristics, or colonization by MDR-GNB or EBSL-producing microorganisms.

A univariate logistic regression analysis was performed in order to identify any association between male gender, age, length of stay in the LTCF, the presence of neurologic disease, diabetes or a urinary catheter, hospitalization or antimicrobial use within the last 3 months and the Charlson comorbidity index with colonization by MDR-GNB and ESBL-producing microorganisms. Then, a multivariate logistic regression analysis with factors identified to have *p* < 0.1 in the univariate analysis was performed. The results of the regression analysis are shown in Table 6. An association between colonization by ESBL-producing microorganisms and male gender, length of stay in the LTCF, presence of a urinary catheter and hospitalization or antimicrobial use within the last three months was identified by the univariate analysis. The multivariate logistic regression analysis identified male gender [odds ratio: 4.358 (1.543–12.313)] and antimicrobial use within the last three months [odds ratio: 5.429 (1.006–29.288)] to be independently positively associated with colonization by ESBL-producing microorganisms. On the other hand, an association between colonization by MDR-GNB and male gender, length of stay in the LTCF, presence of a urinary catheter and antimicrobial use within the last three months was identified by the univariate analysis. The multivariate logistic regression analysis identified male gender [odds ratio: 4.067 (1.415–11.694)] and antimicrobial use within the last three months [odds ratio: 3.985 (1.037–15.323)] to be independently positively associated with colonization by MDR-GNB.

## 4. Discussion

The epidemiology of MDR-GNB among LTCF residents has not been extensively studied, while, to our knowledge, no such study has been conducted in Greece. Thus, this is the first study investigating the epidemiology and risk factors for MDR-GNB colonization in LTCF residents in the Greek population.

MDR-GNB are an increasingly prevalent problem in terms of public health [1]. Infections by such organisms are common among LTCF residents, leading to increased morbidity, mortality and hospital costs [5], while their treatment is problematic, as there are few treatment choices and these choices are often associated with complications [5].

Greece has an increasing prevalence of MDR-GNB and ESBL-producing microorganisms, as shown by a recent study that summarizes the microbiology and antimicrobial susceptibilities of pathogens isolated from the bloodstream of patients in Greek hospitals [19]. Indeed, high rates of carbapenem resistance have been shown during the last 20 years in Greece, with the induction of multiple mechanisms of resistance such as the Verona Integron-encoded Metallo-beta-lactamase (VIM)-producers or Oxacillinase (OXA)-48-carbapenemase that have important implications on mortality [20,21,22,23,24,25,26]. Furthermore, there are studies suggesting that there is an increasing prevalence of infections by MDR-GNB microorganisms in geriatric patients in particular [27]. This makes obvious the necessity for developing countermeasures to reduce the spread of MDR-GNB. To that end, implementation of an antimicrobial stewardship program in different hospitals could reduce the spread of antimicrobial resistance. Hence, restriction of antimicrobial prescription can significantly reduce antimicrobial use and antimicrobial resistance rates, as was shown in a Greek study involving patients in the Intensive Care Unit [28].

Being a LTCF resident is considered to be a risk factor for MDR-GNB colonization, while this increased risk may be associated with the influx of microorganisms from the hospitals to the LTCFs due to frequent hospital visits and hospitalizations of LTCF residents [29]. In terms of infection control, preventing MDR-GNB colonization in LTCFs is difficult, as it requires adequate staff, resources, extensive training, and surveillance [30]. Thus, understanding the mechanism of MDR acquisition is crucial in order to allow adequate infection control in LTCFs. To that direction, a network aiming to perform surveillance of MDR microorganisms through retrospective analysis of antimicrobial susceptibility data obtained in the context of standard clinical care, active rectal swab culturing to determine colonization by ESBL-producing microorganisms or MDR-GNB, as well as surveillance through point-prevalence colonization studies in different LTCFs may lead to better epidemiological surveillance, and infection control, that together with appropriate antimicrobial stewardship measures could lead to reduced rates of colonization by these resistant microorganisms [28,31].

The present study showed that about one in every four LTCF residents in the area of Heraklion is colonized by MDR-GNB. The ESBL phenotype was highly prevalent, with about 25% of LTCF residents harboring microorganisms with that phenotype. This prevalence is comparable to that reported in other studies, where colonization by MDR-GNB is in the range of 11.2% to 59.1% [1,32,33]. Importantly, these high rates of antimicrobial resistance are comparable to the resistance rates noted in hospital acquired infections in Greece [34,35].

In the study’s population, recent antimicrobial use was found to be associated with higher MDR-GNB carriage, which is line with the literature, where recent exposure to antimicrobials and frequent hospitalization are known to be associated with increasing numbers of MDR-GNB in LTCFs [6,32,36]. Even though in the present population, recent hospitalization did not show statistically significant association with MDR-GNB colonization, there was a trend towards higher recent hospitalization in LTCF residents colonized by ESBL-positive microorganisms, which has also been shown in other studies [32,37]. Importantly, a multivariate logistic regression analysis model identified recent antimicrobial use as well as male gender to be the only independent factors associated with an increased likelihood of colonization by MDR-GNB and ESBL-producing microorganisms. Interestingly, the same model did not identify presence of a urinary catheter to be independently associated with a higher likelihood of colonization by MDR-GNB or ESBL-producing microorganisms, even though this had been identified by the contingency analysis to be more prevalent among colonized individuals and was also identified by the univariate analysis as a factor associated with colonization. Thus, the presence of a urinary catheter may be more frequent among individuals colonized by MDR-GNB or ESBL-producing microorganisms, but is not independently associated with this colonization. This is in contrast to other studies suggesting that the presence of a urinary catheter may be associated with an increased risk of colonization by resistant bacteria [6,38,39,40]. In the study population, as was expected, the most common isolated GNB was *E. coli*, both in the MDR- and non-MDR-GNB, which is in line with other studies [4,37].

This study highlights the antimicrobial resistance patterns noted in LTCFs in the Heraklion area on the island of Crete. Thus, it provides valuable information that could have important implications in terms of infection control. The high prevalence of MDR-GNB and ESBL-producing microorganisms in LTCFs implies that individuals admitted in them could be screened for such resistant microorganisms and this could allow isolation of individuals colonized by such pathogens [41]. Furthermore, the results of this study signify the need for increased awareness of healthcare personnel in the hospitals that admit patients residing in LTCFs. Based on these high antimicrobial resistance rates, it is important to provide adequate empirical antimicrobial coverage in patients residing in LTCFs that present with infections possibly caused by these pathogens [42]. Appropriate empirical treatment with extended-spectrum antimicrobials would allow for a reduction in mortality and morbidity in the case of infections by resistant microorganisms, while timely step-down based on culture results in the context of an active antimicrobial stewardship program could aid in the reduction of development of further antimicrobial resistance in hospitals [42]. On the other hand, these results should increase the efforts of antimicrobial stewardship programs in the hospitals, and if possible, in the community and the LTCFs as well, as these antimicrobial resistance patterns noted in LTCF residents are mainly the consequence of antimicrobial overuse and misuse [43,44]. Of note, not all nursing homes had the same colonization rates for MDR-GNB, since two of them had a colonization rate of less than 10%, while, in contrast, one nursing home had an MDR-GNB colonization rate of 100%, even though the number of participants from that facility was very low. This signifies the need for targeted interventions, with infection control measures in different nursing homes based on their individualized colonization profile. To that end, immediate actions should be taken in order to address the problem of extremely high antimicrobial resistance to that specific nursing home.

The present study has some limitations that should be mentioned, such as the relatively small sample size. On the other hand, it was not possible to assess the type of colonization and/or subsequent infection, since one sample was analyzed for each participant. Furthermore, only one rectal swab sample was taken from each participant; thus, the theoretical possibility for errors, for example associated with the performance of swab sampling, exists. Moreover, different pathological conditions, such as Alzheimer’s disease or Parkinson’s disease, were not studied individually, but were pooled during the analysis, so, the effect of each different condition could not be studied. Finally, investigation for MDR-GNB carriage among the personnel of these nursing homes was not performed.

## 5. Conclusions

This regional study shows that nursing homes in the Heraklion area on the island of Crete may be important reservoirs for MDR-GNB transmission. Studies in larger populations are required to confirm the present results that could have important implications on infection control practices and the use of the empirical antimicrobial treatment.

## Figures and Tables

**Table 1:** Isolated microorganisms.

Isolate	# of Isolates	% Among All Isolates
*Escherichia.coli* ESBL positive MDR	164 35 32	64.3 13.7 12.5
*Klebsiella pneumoniae* ESBL positive MDR	30 6 7	11.8 2.4 2.7
*Pseudomonas aeruginosa* MDR	14 1	5.5 0.4
*Proteus mirabilis* ESBL positive MDR	13 1 1	5.1 0.4 0.4
*Comamonas testosteroni*	8	3.1
*Providencia stuartii*	5	2
*Citrobacter koseri*	3	1.2
*Enterobacter cloacae complex*	2	0.8
*Citrobacter freundii*	2	0.8
*Enterobacter aerogenes*	2	0.8
*Morganella morganii*	2	0.8
*Acinetobacter baumanii* MDR	2 1	0.8 0.4
*Klebsiella oxytoca*	1	0.4
*Acinetobacter lwοffii*	1	0.4
*Ochrobactrum anthropi*	1	0.4
*Kluyvera intermedia*	1	0.4
*Aeromonas hydrophila*	1	0.4
*Klebsiella ornitholytica*	1	0.4
*Pantoea spp*	1	0.4
*Aeromonas sobria*	1	0.4

#: number; %: percentage; ESBL: extended-spectrum beta lactamase; MDR: multi-drug resistant.

**Table 2:** Characteristics of LTCF residents regarding hospitalization within the last 3 months.

	Hospitalization within 3 m (n = 7)	No Hospitalization within 3 m (n = 130)	*p*
Male gender, n (%)	1 (14.3)	26 (20)	1
Age, mean (SD)	84.7 (5.3)	82.1 (8.4)	0.4082
Length of LTCF stay, months, median (IQR)	12 (2–70)	22 (10–48)	0.5537
Charlson > or = to 3, n (%)	1 (14.3)	15 (11.5)	0.5895
Diabetes, n (%)	1 (14.3)	26 (20)	1
Neurologic disorder, n (%)	5 (71.4)	103 (79.2)	0.6391
Urinary catheter, n (%)	1 (14.3)	6 (4.6)	0.3132
Antimicrobial use within 3 m, n (%)	6 (85.7)	6 (4.6)	<0.0001
ESBL carriers, n (%)	4 (57.1)	32 (24.6)	0.0775
MDR-GNB carriers, n (%)	3 (42.9)	30 (23.1)	0.3587

n: number; %: percentage; SD: standard deviation; LTCF: long-term care facility; IQR: interquartile range; ESBL: extended-spectrum beta lactamase; MDR-GNB: multi-drug resistant Gram-negative bacteria; *p*: *p*-value.

**Table 3:** Characteristics of LTCF residents regarding antimicrobial use within the last 3 months.

	Antimicrobial Use within 3 m (n = 12)	No Antimicrobial Use within 3 m (n = 125)	*p*
Male gender, n (%)	1 (8.3)	26 (20.8)	0.4595
Age, mean (SD)	83.3 (6.2)	81.9 (8.6)	0.5813
Length of LTCF stay, months, median (IQR)	13.5 (7–30.75)	23 (10–48)	0.2180
Charlson > or = to 3, n (%)	1 (8.3)	15 (12)	1
Diabetes, n (%)	3 (25)	24 (19.2)	0.7039
Neurologic disorder, n (%)	10 (83.3)	98 (78.4)	1
Urinary catheter, n (%)	1 (8.3)	6 (4.8)	0.4815
Hospitalization within 3 m, n (%)	6 (50)	1 (0.8)	<0.0001
ESBL carriers, n (%)	7 (58.3)	29 (23.2)	0.0144
MDR-GNB carriers, n (%)	6 (50)	27 (21.6)	0.0386

n: number; %: percentage; SD: standard deviation; LTCF: long-term care facility; IQR: interquartile range; ESBL: extended-spectrum beta lactamase; MDR-GNB: multi-drug resistant Gram-negative bacteria; *p*: *p*-value.

**Table 4:** Characteristics of LTCF residents regarding colonization by MDR-GNB.

	MDR-GNB Carriers (n = 33)	No MDR-GNB Carriers (n = 104)	*p*
Male gender, n (%)	10 (30.3)	17 (16.3)	0.1294
Age, mean (SD)	83.2 (5.9)	81.7 (9)	0.3511
Length of LTCF stay, months, median (IQR)	13 (8.5–29.5)	24 (10–48)	0.0771
Charlson > or = to 3, n (%)	3 (9.1)	13 (12.5)	0.7610
Diabetes, n (%)	4 (12.1)	23 (22.1)	0.3147
Neurologic disorder, n (%)	27 (81.8)	81 (77.9)	0.8076
Urinary catheter, n (%)	7 (21.2)	0 (0)	<0.0001
Hospitalization within 3 m, n (%)	3 (9.1)	4 (3.8)	0.3587
Antimicrobial use within 3 m, n (%)	6 (18.2)	6 (5.8)	0.0386
ESBL carriers, n (%)	33 (100)	3 (2.9)	<0.0001

n: number; %: percentage; SD: standard deviation; LTCF: long-term care facility; IQR: interquartile range; ESBL: extended-spectrum beta lactamase; MDR-GNB: multi-drug resistant Gram-negative bacteria; *p*: *p*-value.

**Table 5:** Characteristics of LTCF residents regarding colonization by ESBL-producing microorganisms.

	ESBL Carriers (n = 36)	No ESBL Carriers (n = 101)	*p*
Male gender, n (%)	11 (30.6)	16 (15.8)	0.0855
Age, mean (SD)	83.7 (6.6)	81.5 (8.8)	0.197
Length of LTCF stay, months, median (IQR)	13 (8.5–29.5)	24 (10–48)	0.0834
Charlson > or = to 3, n (%)	3 (8.3)	13 (12.9)	0.5603
Diabetes, n (%)	4 (11.1)	23 (22.7)	0.1509
Neurologic disorder, n (%)	30 (83.3)	78 (77.2)	0.4879
Urinary catheter, n (%)	7 (19.4)	0 (0)	<0.0001
Hospitalization within 3 m, n (%)	4 (11.1)	3 (3)	0.0775
Antimicrobial use within 3 m, n (%)	7 (19.4)	5 (5)	0.0144
MDR-GNB carriers, n (%)	33 (91.7)	0 (0)	<0.0001

n: number; %: percentage; SD: standard deviation; LTCF: long-term care facility; IQR: interquartile range; ESBL: extended-spectrum beta lactamase; MDR-GNB: multi-drug resistant Gram-negative bacteria; *p*: *p*-value.

**Table 6:** Regression analysis of colonization of LTCF residents by ESBL-producing microorganisms or MDR-GNB.

ESBL	Univariate Analysis *p*	Multivariate Analysis *p*	OR (95% CI)
Male gender	0.0573	0.005	4.358 (1.543–12.313)
Length of stay in LTCF	0.0775	0.066	0.985 (0.97–1.001)
Urinary catheter	<0.0001	0.999	6.356 × 10^9^ (0.000–infinity)
Hospitalization within 3 months	0.0574	0.975	0.962 (0.088–10.537)
Antimicrobial use within 3 months	0.008	0.049	5.429 (1.006–29.288)
**MDR-GNB**	**Univariate Analysis** ***p***	**Multivariate Analysis** ***p***	**OR (95% CI)**
Male gender	0.0801	0.009	4.067 (1.415–11.694)
Length of stay in LTCF	0.0771	0.063	0.984 (0.968–1.001)
Urinary catheter	<0.0001	0.999	7.297 × 10^9^ (0.000–infinity)
Antimicrobial use within 3 months	0.028	0.044	3.985 (1.037–15.323)

CI: confidence interval; LTCF: long-term care facility; ESBL: extended-spectrum beta lactamase; MDR-GNB: multi-drug resistant Gram-negative bacteria; OR: odds ratio; *p*: *p*-value.

## Data Availability

The data presented in this study are available on request from the corresponding author.

## Supplementary Materials

The following are available online at https://www.mdpi.com/article/10.3390/tropicalmed6030123/s1, Table S1: Characteristics of LTCF residents according to gender.

## References

[1] Aliyu S., Smaldone A., Larson E. (2017). Prevalence of Multidrug-Resistant Gram-Negative Bacteria among Nursing Home Residents: A Systematic Review and Meta-Analysis. Am. J. Infect. Control..

[2] Cillóniz C., Dominedò C., Torres A. (2019). Multidrug Resistant Gram-Negative Bacteria in Community-Acquired Pneumonia. Crit. Care.

[3] Cosgrove S.E. (2006). The Relationship between Antimicrobial Resistance and Patient Outcomes: Mortality, Length of Hospital Stay, and Health Care Costs. Clin. Infect. Dis.

[4] Tseng W.-P., Chen Y.-C., Yang B.-J., Chen S.-Y., Lin J.-J., Huang Y.-H., Fu C.-M., Chang S.-C., Chen S.-Y. (2017). Predicting Multidrug-Resistant Gram-Negative Bacterial Colonization and Associated Infection on Hospital Admission. Infect. Control. Hosp. Epidemiol..

[5] Kallen A.J., Srinivasan A. (2010). Current Epidemiology of Multidrug-resistant Gram-negative Bacilli in the United States. Infect. Control. Hosp. Epidemiol.

[6] Flokas M.E., Alevizakos M., Shehadeh F., Andreatos N., Mylonakis E. (2017). Extended-Spectrum β-Lactamase-Producing Enterobacteriaceae Colonisation in Long-Term Care Facilities: A Systematic Review and Meta-Analysis. Int. J. Antimicrob. Agents.

[7] Ludden C., Cormican M., Vellinga A., Johnson J.R., Austin B., Morris D. (2015). Colonisation with ESBL-Producing and Carbapenemase-Producing Enterobacteriaceae, Vancomycin-Resistant Enterococci, and Meticillin-Resistant Staphylococcus Aureus in a Long-Term Care Facility over One Year. BMC Infect. Dis..

[8] March A., Aschbacher R., Sleghel F., Soelva G., Kaczor M., Migliavacca R., Piazza A., Mattioni Marchetti V., Pagani L., Scalzo K. (2017). Colonization of Residents and Staff of an Italian Long-Term Care Facility and an Adjacent Acute Care Hospital Geriatric Unit by Multidrug-Resistant Bacteria. New Microbiol..

[9] van Dulm E., Tholen A.T.R., Pettersson A., van Rooijen M.S., Willemsen I., Molenaar P., Damen M., Gruteke P., Oostvogel P., Kuijper E.J. (2019). High Prevalence of Multidrug Resistant Enterobacteriaceae among Residents of Long Term Care Facilities in Amsterdam, the Netherlands. PLoS ONE.

[10] Hogardt M., Proba P., Mischler D., Cuny C., Kempf V.A., Heudorf U. (2015). Current Prevalence of Multidrug-Resistant Organisms in Long-Term Care Facilities in the Rhine-Main District, Germany, 2013. Euro Surveill.

[11] McKinnell J.A., Singh R.D., Miller L.G., Kleinman K., Gussin G., He J., Saavedra R., Dutciuc T.D., Estevez M., Chang J. (2019). The SHIELD Orange County Project: Multidrug-Resistant Organism Prevalence in 21 Nursing Homes and Long-Term Acute Care Facilities in Southern California. Clin. Infect. Dis..

[12] Tinelli M., Tiseo G., Falcone M., ESCMID Study Group for Infections in the Elderly (2021). Prevention of the Spread of Multidrug-Resistant Organisms in Nursing Homes. Aging Clin. Exp. Res..

[13] Rosello A., Horner C., Hopkins S., Hayward A.C., Deeny S.R. (2017). Understanding the Impact of Interventions to Prevent Antimicrobial Resistant Infections in the Long-Term Care Facility: A Review and Practical Guide to Mathematical Modeling. Infect. Control. Hosp. Epidemiol..

[14] Moro M.L., Gagliotti C. (2013). Antimicrobial Resistance and Stewardship in Long-Term Care Settings. Future Microbiol..

[15] Magiorakos A.-P., Srinivasan A., Carey R.B., Carmeli Y., Falagas M.E., Giske C.G., Harbarth S., Hindler J.F., Kahlmeter G., Olsson-Liljequist B. (2012). Multidrug-Resistant, Extensively Drug-Resistant and Pandrug-Resistant Bacteria: An International Expert Proposal for Interim Standard Definitions for Acquired Resistance. Clin. Microbiol. Infect..

[16] Clinical and Laboratory Standards Institute (2017). Performance Standards for Antimicrobial Susceptibility Testing.

[17] (2010). Food and Drug Administration (FDA) Prescribing Information for Tygacil (Tigecycline). Http://Www.Accessdata.Fda.Gov/Drugsatfda_docs/Label/2010/021821s021lbl.Pdf.

[18] The European Committee on Antimicrobial Susceptibility Testing (2017). Breakpoint Tables for Interpretation of MICs and Zone Diameters (v. 7.1). Http://Www.Eucast.Org.

[19] Polemis M., Tryfinopoulou K., Giakkoupi P., Vatopoulos A., WHONET-Greece Study Group (2020). Eight-Year Trends in the Relative Isolation Frequency and Antimicrobial Susceptibility among Bloodstream Isolates from Greek Hospitals: Data from the Greek Electronic System for the Surveillance of Antimicrobial Resistance—WHONET-Greece, 2010 to 2017. Euro Surveill.

[20] Grundmann H., Glasner C., Albiger B., Aanensen D.M., Tomlinson C.T., Andrasević A.T., Cantón R., Carmeli Y., Friedrich A.W., Giske C.G. (2017). Occurrence of Carbapenemase-Producing Klebsiella Pneumoniae and Escherichia Coli in the European Survey of Carbapenemase-Producing Enterobacteriaceae (EuSCAPE): A Prospective, Multinational Study. Lancet Infect. Dis..

[21] Vatopoulos A. (2008). High Rates of Metallo-Beta-Lactamase-Producing Klebsiella Pneumoniae in Greece--A Review of the Current Evidence. Euro Surveill.

[22] Maltezou H.C., Giakkoupi P., Maragos A., Bolikas M., Raftopoulos V., Papahatzaki H., Vrouhos G., Liakou V., Vatopoulos A.C. (2009). Outbreak of Infections Due to KPC-2-Producing Klebsiella Pneumoniae in a Hospital in Crete (Greece). J. Infect..

[23] Voulgari E., Gartzonika C., Vrioni G., Politi L., Priavali E., Levidiotou-Stefanou S., Tsakris A. (2014). The Balkan Region: NDM-1-Producing Klebsiella Pneumoniae ST11 Clonal Strain Causing Outbreaks in Greece. J. Antimicrob. Chemother..

[24] Mavroidi A., Miriagou V., Malli E., Stefos A., Dalekos G.N., Tzouvelekis L.S., Petinaki E. (2012). Emergence of Escherichia Coli Sequence Type 410 (ST410) with KPC-2 β-Lactamase. Int. J. Antimicrob. Agents..

[25] Tsakris A., Poulou A., Bogaerts P., Dimitroulia E., Pournaras S., Glupczynski Y. (2015). Evaluation of a New Phenotypic OXA-48 Disk Test for Differentiation of OXA-48 Carbapenemase-Producing Enterobacteriaceae Clinical Isolates. J. Clin. Microbiol..

[26] Cassini A., Högberg L.D., Plachouras D., Quattrocchi A., Hoxha A., Simonsen G.S., Colomb-Cotinat M., Kretzschmar M.E., Devleesschauwer B., Cecchini M. (2019). Attributable Deaths and Disability-Adjusted Life-Years Caused by Infections with Antibiotic-Resistant Bacteria in the EU and the European Economic Area in 2015: A Population-Level Modelling Analysis. Lancet Infect. Dis..

[27] Ioannou P., Plexousaki M., Dimogerontas K., Aftzi V., Drougkaki M., Konidaki M., Paschalidis K., Maraki S., Kofteridis D.P. (2020). Characteristics of Urinary Tract Infections in Older Patients in a Tertiary Hospital in Greece. Geriatr. Gerontol. Int..

[28] Ntagiopoulos P.G., Paramythiotou E., Antoniadou A., Giamarellou H., Karabinis A. (2007). Impact of an Antibiotic Restriction Policy on the Antibiotic Resistance Patterns of Gram-Negative Microorganisms in an Intensive Care Unit in Greece. Int. J. Antimicrob. Agents.

[29] Cerceo E., Deitelzweig S.B., Sherman B.M., Amin A.N. (2016). Multidrug-Resistant Gram-Negative Bacterial Infections in the Hospital Setting: Overview, Implications for Clinical Practice, and Emerging Treatment Options. Microb. Drug Resist..

[30] Mody L., Bradley S.F., Galecki A., Olmsted R.N., Fitzgerald J.T., Kauffman C.A., Saint S., Krein S.L. (2011). Conceptual Model for Reducing Infections and Antimicrobial Resistance in Skilled Nursing Facilities: Focusing on Residents with Indwelling Devices. Clin. Infect. Dis..

[31] Aschbacher R., Pagani L., Migliavacca R., Pagani L. (2020). GLISTer (Gruppo di Lavoro per lo Studio delle Infezioni nelle Residenze Sanitarie Assistite e Strutture Assimilabili) working group Recommendations for the Surveillance of Multidrug-Resistant Bacteria in Italian Long-Term Care Facilities by the GLISTer Working Group of the Italian Association of Clinical Microbiologists (AMCLI). Antimicrob. Resist. Infect. Control..

[32] Gruber I., Heudorf U., Werner G., Pfeifer Y., Imirzalioglu C., Ackermann H., Brandt C., Besier S., Wichelhaus T.A. (2013). Multidrug-Resistant Bacteria in Geriatric Clinics, Nursing Homes, and Ambulant Care--Prevalence and Risk Factors. Int. J. Med. Microbiol..

[33] Marchaim D., Chopra T., Bogan C., Bheemreddy S., Sengstock D., Jagarlamudi R., Malani A., Lemanek L., Moshos J., Lephart P.R. (2012). The Burden of Multidrug-Resistant Organisms on Tertiary Hospitals Posed by Patients with Recent Stays in Long-Term Acute Care Facilities. Am. J. Infect. Control..

[34] Geladari A., Karampatakis T., Antachopoulos C., Iosifidis E., Tsiatsiou O., Politi L., Karyoti A., Papanikolaou V., Tsakris A., Roilides E. (2017). Epidemiological Surveillance of Multidrug-Resistant Gram-Negative Bacteria in a Solid Organ Transplantation Department. Transpl. Infect. Dis..

[35] Miyakis S., Pefanis A., Tsakris A. (2011). The Challenges of Antimicrobial Drug Resistance in Greece. Clin. Infect. Dis..

[36] Kahvecioglu D., Ramiah K., McMaughan D., Garfinkel S., McSorley V.E., Nguyen Q.N., Yang M., Pugliese C., Mehr D., Phillips C.D. (2014). Multidrug-Resistant Organism Infections in US Nursing Homes: A National Study of Prevalence, Onset, and Transmission across Care Settings, 1 October 2010–31 December 2011. Infect. Control. Hosp. Epidemiol..

[37] Giufrè M., Ricchizzi E., Accogli M., Barbanti F., Monaco M., Pimentel de Araujo F., Farina C., Fazii P., Mattei R., Sarti M. (2017). Colonization by Multidrug-Resistant Organisms in Long-Term Care Facilities in Italy: A Point-Prevalence Study. Clin. Microbiol. Infect..

[38] Jans B., Schoevaerdts D., Huang T.-D., Berhin C., Latour K., Bogaerts P., Nonhoff C., Denis O., Catry B., Glupczynski Y. (2013). Epidemiology of Multidrug-Resistant Microorganisms among Nursing Home Residents in Belgium. PLoS ONE.

[39] Rooney P.J., O’Leary M.C., Loughrey A.C., McCalmont M., Smyth B., Donaghy P., Badri M., Woodford N., Karisik E., Livermore D.M. (2009). Nursing Homes as a Reservoir of Extended-Spectrum Beta-Lactamase (ESBL)-Producing Ciprofloxacin-Resistant Escherichia Coli. J. Antimicrob. Chemother..

[40] Andersson H., Lindholm C., Iversen A., Giske C.G., Örtqvist Å., Kalin M., Fossum B. (2012). Prevalence of Antibiotic-Resistant Bacteria in Residents of Nursing Homes in a Swedish Municipality: Healthcare Staff Knowledge of and Adherence to Principles of Basic Infection Prevention. Scand. J. Infect. Dis..

[41] Cassone M., Mody L. (2015). Colonization with Multi-Drug Resistant Organisms in Nursing Homes: Scope, Importance, and Management. Curr. Geriatr. Rep..

[42] Aliyu S., McGowan K., Hussain D., Kanawati L., Ruiz M., Yohannes S. (2021). Prevalence and Outcomes of Multi-Drug Resistant Blood Stream Infections Among Nursing Home Residents Admitted to an Acute Care Hospital. J. Intensive Care Med..

[43] Kruger S.Z., Bronskill S.E., Jeffs L., Steinberg M., Morris A.M., Bell C.M. (2020). Evaluating and Prioritizing Antimicrobial Stewardship Programs for Nursing Homes: A Modified Delphi Panel. Infect. Control. Hosp. Epidemiol..

[44] Agarwal M., Dick A.W., Sorbero M., Mody L., Stone P.W. (2020). Changes in US Nursing Home Infection Prevention and Control Programs From 2014 to 2018. J. Am. Med. Dir. Assoc..

