# Supplementary material for: Rectal Colonization by Drug Resistant Bacteria in Nursing Home Residents in Crete, Greece
Source: Trop Med Infect Dis. 2021 Jul 5;6(3):123. doi: 10.3390/tropicalmed6030123 (PMC8293340; doi:10.3390/tropicalmed6030123)
Supplement: Supplementary file 1 [file tropicalmed-06-00123-s001.zip › tropicalmed-1239461-supplementary.pdf]

Tropical Medicine and Infectious Disease

MDPI

Supplementary

Table S1. Characteristics of LTCF residents according to gender.

	**Male (n = 27)**	**Female (n = 110)**	***p***
Age, mean (SD)	80 (9.3)	82.6 (8.1)	0.1648
Length of LTCF stay, months, median (IQR)	36 (11-81)	20 (9-43.8)	0.0118
Charlson > or = to 3, n (%)	5 (18.5)	11 (10)	0.1747
Diabetes, n (%)	3 (11.1)	24 (21.8)	0.2844
Neurologic disorder, n (%)	20 (74.1)	88 (80)	0.5991
Urinary catheter, n (%)	1 (3.7)	6 (5.5)	1
Hospitalization within 3m, n (%)	1 (3.7)	6 (5.5)	1
Antibiotic use within 3m, n (%)	1 (3.7)	11 (10)	0.4595
ESBL carriers, n (%)	11 (40.7)	25 (22.7)	0.0855
MDR-GNB carriers, n (%)	10 (37)	23 (20.9)	0.1294

n: number; %: percentage; SD: standard deviation; LTCF: long-term care facility; IQR: interquartile range; ESBL: extended-spectrum beta lactamase; MDR-GNB: multi-drug resistant gram-negative bacteria; p: p-value.
